# Diagnostic performance and predictive value of rheumatoid factor, anti-cyclic-citrullinated peptide antibodies and HLA-DRB1 locus genes in rheumatoid arthritis

**DOI:** 10.1186/1755-7682-1-20

**Published:** 2008-10-22

**Authors:** Nihal A Fathi, Azza M Ezz-Eldin, Eman Mosad, Rania M Bakry, Hosny B Hamed, Sahar Ahmed, Marwa Mahmoud, Hebat-Allah G Rashed, Fatma Abdullah

**Affiliations:** 1 Rheumatology department, Assiut University Hospital, Assiut, Egypt; 2 Clinical pathology department, Assiut University Hospital, Assiut, Egypt; 3 HLA Unit, Clinical pathology department, South Egypt Cancer Institute, Assiut University, Assiut, Egypt

## Abstract

**Background:**

We evaluated the significance of the genes, defined as DRB1*04 or DRB1*01, in rheumatoid arthritis (RA) patients. We focused on the role of genetic and serologic markers to predict disease activity and destructive process of joints.

**Methods:**

Sixty patients with RA were examined. Radiographic changes were evaluated by (Larsen score) and disease activity was measured by disease activity score 28 (DAS28). The markers analyzed were: erythrocyte sedimentation rate (ESR), C-reactive protein (CRP), rheumatoid factor (RF), anti-cyclic citrullinated peptides (anti-CCP2) and HLA-*DRB1 *alleles typed by PCR.

**Results:**

In this study, anti-CCP antibodies, CRP, RF and AKA were detected in 83.3%, 56.7%, 71.7% and 52% of patients respectively. HLA-DRB1*01 was found in 45% of patients and 35% of them had one or two HLA-DRB1*04 alleles. According to DRB1*04 subtypes, (DRB1* 0405) was present in of 80% them. For prediction of grade of activity, the independent predictors were anti-CCP (OR 19.6), and DRB1*04 positive allele (OR 5.1). The combination of DRB1*04 + anti-CCP antibodies gave increase in the specificity and positive predictive value to 92% and 90 respectively. As regards to the prediction of radiological joint damage, the independent predictors were HLA-DRB1*04, HLA-DRB1*01, RF, and CRP > 18 (OR 5.5, 4.5, 2.5, 2.0 respectively).

**Conclusion:**

Our findings suggest that anti-CCP2 is superior to RF for the detection of RA and provided predictive information on joint destruction and disease activity. The presence of RA associated antibodies (ACCP or RF) and/or the SE genes are indicative for a poorer radiological outcome and higher grade of activity.

## Introduction

Rheumatoid arthritis (RA) is an inflammatory disease of unknown cause. The course of rheumatoid arthritis is ranging from mild to aggressive forms, the latter being very difficult to cope with. It has been shown that early diagnosis and treatment reduce joint destruction, and improve survival [1]. Risk factors have been identified in groups of patients with different outcomes such as baseline radiographic joint changes, presence of rheumatoid factor (RF), specific human leukocyte antigens (HLA); HLA-*DRB1 *genotypes, high disease activity, high disability scores, and high levels of acute phase proteins [2]. Antibodies to cyclic citrullinated peptide (anti-CCP) have been documented extensively over recent years as highly specific serological markers for rheumatoid arthritis, with important clinical implications for diagnosis and prognosis [3].

It has been suggested that the etiology of RA is due to an interaction between genetic and environmental factors. Genetic studies have demonstrated multiple HLA-*DRB1 *alleles encoding a conserved sequence at amino acid positions 70–74 are associated with susceptibility and severity of RA. This conserved sequence is commonly known as the shared epitope (SE). The role of the SE in the evolution of articular destruction has yielded conflicting results [4,5].

The present study is a cross-sectional analysis aimed to evaluate the significance of the presence of SE genes, defined as DRB1*01 or DRB1*04, in relation to anti-CCP antibodies, antikeratin antibody (AKA) and RF in individuals who developed RA. We focused on disease activity and joint damage, evaluated on radiographs, as outcome variables.

## Methods

This study was carried out on 60 outpatients who fulfilled the American college of rheumatology criteria for RA. A written consent was obtained from the patients according to the Declaration of Helsinki prior to enrollment in the study. The study was approved by the Assiut University local ethical committee. Patients were subsequently treated with disease modifying antirheumatic drugs (usually methotrexate, sulphasalazine, or a combination of both). The patients were evaluated for age; sex; body mass index; disease duration; duration of morning stiffness; patients' assessment of pain (on a visual analogue scale); number of swollen and tender joints, disease activity score; presence or absence of nodules and extra-articular manifestations. Disease activity by the disease activity score (DAS28) [6]. Global health and pain and visual analogue scale were assessed. Functional disability was evaluated using health assessment questionnaire [7]. Hand, wrist, and foot radiographs were obtained, evaluated and scored using Larsen method [8].

### Sample handling and investigations

Blood samples were collected; for complete blood count, erythrocyte sedimentation rate (ESR), C-reactive protein (CRP) and DNA isolation. One hundred samples were taken from healthy blood donors as a control for HLA typing; twenty of them were used for serologic markers.

### Determination of antibodies

RF was detected by the kit supplied Biotec Laboratories Lot No. RF -460 based on agglutination test using particles sensitized with human IgG. ANA was detected by the Fluro-kit supplies by supplied by DiaSorine Catalog No 1740 based on indirect immunoflorescent for the screening and titration of antinuclear antibodies (ANA). Anti-CCP2 antibodies were detected by enzyme-linked immunosorbent assay Kit supplies by INOVA Diagnostica Cat. NO 570139. Serum samples with a test result 25 U/ml were considered positive, and designated as the "standard" cut off. AKA was detected by kit supplied by IMMECO Diagnostic, Lot No. 2120-8, based on indirect immunofluorescence antibodies on rat esophagus substrate.

### HLA-DRB1 genotyping

HLA typing was performed using sequence specific oligonucleotide technique, reversed dot blot hybridization technique (Dynal RELI HLA-*DRB1*, product No.802.01) [9]. DNA for *HLA-DRB1 *typing of patients was isolated from peripheral blood mononuclear cells by using the DNA Isolation Kit (QIAamp minikit, Qiagen, Hilden, Germany). The biotinylated primers anneal to their target, Tag DNA polymerase extend the annealed primers along the target templates to produce a biotinylated DNA sequence (amplicon). After polymerase chain reaction amplification, the amplicons are chemically denatureated, theses are added to nylon membrane that contains an array of immobilized sequence specific oligonucleotide probes that contain a complementary target sequence and thus are captured onto the membrane strip. The amplicon-probe complex is visualized using a colorimetric reaction. The resulting probe signals are captured to the control probe intensity and the samples recoded for interpretation [10]. The specificity of the amplification can be locus specific (eg, HLA-A, HLA-B, HLA-*DRB1*) group specific (eg, DRB1*01, DRB1*02), or allele-specific (eg, *DRB1-0404*, *DRB1-0405*). According to HLA-DRB1*04 subtyping the shared epitopes encoding alleles *0409,*0413, *0416 and *0421 were classified as *0401 alleles, *0408, *0410 and *0419 were classified as *0404. The DRB1*0405 alleles were analyzed as a separate entity. All other DRB1*04 alleles were classified as DRB1*04 non-SE alleles [4].

### Statistical analysis

Statistical analyses were done using SPSS 15 statistical software (SPSS Inc, Chicago, Illinois, USA). Continuous variables were transformed into categorical variables using the median value as the cut off point. Comparison of categorical variables between groups, as well as univariate analysis of the relation between all baseline values and the outcome measure was undertaken using the X^2 ^test, with Fisher's exact correction when appropriate. Sensitivity and specificity, and negative (NPV) and positive (PPV) predictive values of the possible predictors were calculated. Significant univariate variables were included in a multivariate stepwise logistic regression model to find relevant independent prognostic variables. *P *value < 0.05 is considered significant.

## Results

Clinical and laboratory investigations were illustrated in Table 1. The median age of the patients was 50 years and 90% were women. According the positivity of anti-CCP antibodies, 50 (83.3%) patients were anti-CCP antibodies positive. CRP, RF and AKA were detected in 34 (56.7%), 43 (71.7%) and 31 (52%) of patients respectively.

**Table 1:** Clinical and laboratory characteristics of patients

		All patients	Anti-CCP		*P *value
			Negative	Positive	
Age	<50*	45 (75%)	8 (17.8%)	37 (82.2%)	NS
	≥50	15 (25%)	2 (13.3%)	13 (86.7%)	
Sex	female	54 (90%)	9 (16.7%)	45 (83.3%)	NS
	male	6 (10%)	1 (16.7%)	5 (83.3%)	
Duration	<6m	3 (5%)	1 (33.3%)	2 (66.7%)	NS
	≥6m	57 (95%)	9 (15.8%)	48 (84.2%)	
Smoking	yes	5 (8.3%)	1 (20%)	4 (80%)	NS
	no	55 (91.7%)	9 (16.4%)	46 (83.6%)	
CRP	<18 *	26 (43.3%)	7 (26.9%)	19 (73.1%)	NS
	≥18	34 (56.7%)	3 (8.8%)	31 (91.2%)	
RF	+ve	43 (71.7%)	4 (9.3%)	39 (90.7%)	0.02
			6 (35%)	11 (65%)	
ESR-1	<32 *	14 (23.3%)	5 (35.7%)	9 (64.3%)	0.04
	≥32	46 (76.7%)	5 (10.9%)	41 (89.1%)	
ANA	+ve	22 (36.7%)	0 (0%)	22 (100%)	0.03
	-ve	38 (63.3%)	10 (40.8%)	28 (59.2%)	
AKA	+ve	31 (52%)	3 (10%)	28 (90%)	
	-ve	29 (48%)	17 (59%)	12 (41%)	0.05
Grade of activity (DAS)					
- Mild or moderete		25 (41.7%)	9 (31.6%)	16 (68.4%)	0.001
- Severe		35 (58.3%)	1 (9.8%)	34 (90.2%)	
Larsen score	<35*	19 (31.7%)	6 (10%)	13 (21.7%)	0.05
	≥35	41(68.3%)	4 (6.7%)	37 (61.7%)	
HLA-DRB1*04	+ve	21 (35%)	2 (9.5%)	19 (90.5%)	NS
	-ve	39 (65%)	8 (20.5%)	31 (79.5%)	
HLA-RB1*01	+ve	27 (45%)	5 (19%)	22 (81%)	NS
	-ve	33 (55%)	5 (15%)	28 (85%)	

Values are expressed as n (%). AKA: antikeratin antibody; anti-CCP: anti-cyclic citrullinated peptides antibodies; ANA: antinuclear antibodies; DAS: disease activity score; CRP: C-reactive protein; RF: rheumatoid factor; HLA: human leukocyte antigen. * Cut-off values are medians.

Comparing anti-CCP positive with anti-CCP negative patients (Table 1); anti-CCP positive patients had a significantly higher frequency of high ESR, RF, ANA and AKA (*P *= 0.04, 0.02, 0.03 and 0.05) respectively.

The frequencies of HLA-*DRB1 *alleles in patients and control are shown in Table 2. There was a statistically significant increase in the frequency in DRB1*01, DRB1*02, DRB1*04 compared to control (*P *= 0.001, 0.05 and 005 respectively). In this study 45% had HLA-DRB1*01 and 35% of patients had one or two HLA-DRB1*04 alleles.

**Table 2:** Frequencies of HLA-*DRB1 *alleles in patients and control

HLA-*DRB1 *allele	Patients	Control	*P *value
HLA-*DRB1-01*	27(45%)	5(5%)	0.001
HLA-*DRB1-02*	11(18%)	0(0%)	0.05
HLA-*DRB1-03*	10(16%)	50(50%)	0.03
HLA-DRB1-04*	21(35%)	10(10%)	0.05
- HLA-*DRB1-0401*	3(15%)	3(13%)	
- HLA-*DRB1-0404*	1(5%)	9(9%)	
- HLA-*DRB1-0405*	17(80%)	77(77%)	
DRB1*04 non-SE alleles	0 (0%)	1(1%)	
HLA-*DRB1-07*	4(6%)	25(25%)	0.02
HLA-*DRB1-08*	6(10%)	5(5%)	NS
HLA-*DRB-09*	1(1.7%)	1(1%)	NS
HLA-*DRB1-10*	0(0%)	15(15%)	0.002
HLA-*DRB1-11*	5(8.3%)	20(20%)	NS
HLA-*DRB-12*	10(16%)	16(16%)	NS
HLA-*DRB-13*	16(26.7%)	30(30%)	NS
HLA-*DRB-14*	1(1.7%)	4(4%)	NS
HLA-*DRB1-15*	0(0%)	16(16%)	0.002

Values are expressed as n (%). HLA: human leukocyte antigen. *According to HLA-DRB1*04 subtyping the shared epitopes encoding alleles *0409, *0413, *0416 and *0421 were classified as *0401 alleles, *0408, *0410 and *0419 were classified as *0404. The DRB1*0405 alleles were analyzed as a separate entity. All other DRB1*04 alleles were classified as DRB1*04 non-SE alleles.^(4)^

It was found that RF associated allele (DRB1* 0405) was present in of 80% of DRB1*04 positive patients. "Double dose" of DRB1*04 (homozygous) was found in 8/60 (13%) of our patients versus 3/100 (3%) in the control *P *= 0.05 (Data is not shown in the table).

### Predictors of the disease activity grade

Univariate predictors of the grade of disease activity were summarized in Table 3. Anti-CCP had the highest sensitivity; 97%, and NPV 90%. On multivariate analysis, the independent predictors of disease activity were anti-CCP (OR 19.6), and DRB1*04 positive allele (OR 5.1). (Table 4) The combination of DRB1*04 + anti-CCP antibodies gave increase in the specificity and PPV to 92% and 90%, with a sensitivity and NPV of 42% and 41% respectively. The presence of single and double gene carriage of the HLA-DRB1*04 resulted in a significantly higher grade of disease activity (*P *= 0.009) compared with its absence. With regard to a possible gene dosage effect, no statistically significant difference between double and single gene carriage of the DRB1*04 was observed (data is not shown in tables).

**Table 3:** Univariate predictors of disease activity grade

	Sensitivity	Specificity	PPV	NPV	*P *value
Anti-CCP present	97%	36%	68%	90%	0.001
RF present	77%	36%	63%	53%	NS
CRP > 18	37%	72%	100%	51%	0.002
ESR1 > 32	89%	40%	67%	71%	0.014
ANA	20%	68%	51%	45%	NS
AKA	65%	68%	74%	59%	0.01
Age > 50	29%	80%	67%	44%	NS
Duration > 6m	97%	8%	60%	67%	NS
Smoking	9%	92%	60%	42%	NS
Larsen score ≥35	80%	48%	68%	63%	0.02
HLA-DRB1*01	40%	48%	51%	36%	NS
HLA-DRB1*04	48%	84%	81%	54%	0.009

AKA: antikeratin antibody; anti-CCP: anti-cyclic citrullinated peptides antibodies; ANA: antinuclear antibodies; CRP: C-reactive protein; HLA: human leukocyte antigen; NPV: negative predictive value; PPV: positive predictive value; RF: rheumatoid factor

**Table 4:** Multivariate predictors of disease activity grade

	OR (95%CI)	*P *value
Anti-CCP present	19.6 (2.1–48.5)	0.009
HLA-DRB1*04	5.1 (1.2–21.1)	0.03

Anti-CCP: anti-cyclic citrullinated peptides antibodies; HLA: human leukocyte antigen, OR: Odds ratio; CI: confidence interval.

### Predictors of radiological joint damage

ESR first hour > 32 had the highest sensitivity 98%, and NPV 93% (Table 5). In multivariate analysis, the independent predictors were HLA-DRB1*04, HLA-DRB1*01, CRP > 18, and RF (Table 6).

**Table 5:** Univariate predictors of radiological joint damage (Larsen score)

	Sensitivity	Specificity	PPV	NPV	*P *value
Anti-CCP present	90%	32%	74%	60%	0.05
RF present	81%	47%	77%	53%	0.03
CRP > 18	66%	63%	79%	46%	0.05
ESR1 > 32	98%	68%	87%	93%	0.0001
ANA	21%	74%	73%	37%	NS
AKA	56%	58%	74%	38%	NS
Age > 50	32%	90%	86%	38%	NS
Duration > 6m	98%	11%	70%	67%	NS
Risk factor	7%	90%	60%	31%	NS
HLA-DRB1*01	53%	74%	81%	42%	0.05
HLA-DRB1*04	44%	84%	86%	54%	0.04

AKA: antikeratin antibody; anti-CCP: anti-cyclic citrullinated peptides antibodies; ANA: antinuclear antibodies; CRP: C-reactive protein; HLA: human leukocyte antigen; NPV: negative predictive value; PPV: positive predictive value; RF: rheumatoid factor.

**Table 6:** Multivariate predictors of radiological joint damage (Larsen score)

	OR (95%CI)	*P *value
RF present	2.5 (1.1–5.9)	0.01
CRP > 18	2.0 (0.2–5.3)	0.01
HLA-DRB1*01	4.5 (0.4–7.8)	0.002
HLA-DRB1*04	5.5 (0.4–8.7)	0.003

CRP: C-reactive protein; RF: rheumatoid factor; HLA: human leukocyte antigen; OR: Odds ratio; CI: confidence interval.

## Discussion

Anti-CCP-antibody is a synthetic cyclic peptides containing citrulline. It is incorporated into newly proposed diagnostic criteria for rheumatoid arthritis, with higher sensitivity. The high predictive value of anti-CCP antibodies is indicating that citrullination and production of anti-CCP antibodies and RF are early processes in the development of RA [11]. The present work revealed strong complementarities of RF to anti-CCP as 65% patients lacking RF found to be positive for anti-CCP. Some reports suggest that RF and anti-CCP should be combined to reach optimum diagnostic properties [12]. Discrepancy can be explained by the fact that in the literature stratification for specificity was not performed, and therefore, the results of different studies are not comparable [13]. The frequencies of ANA and AKA were 36%, 52% respectively which indicate that testing for this spectrum of auto antibodies carries little advantage over RF or anti-CCP in diagnosing RA. A significant association of AKA with Anti-CCP may relate to that citrulline (profilaggrin) appears to be an essential constituent of the antigenic determinants recognized by AKA [14].

In the present work, the sensitivity of anti-CCP was significantly higher than that of the RFs for prediction of grade of disease activity. This findings were supported by the data previously published [1,15].

It was reported that anti-CCP is an important predictor of radiological outcome. Preventing and diminishing joint damage is an important treatment goal in early rheumatoid arthritis [1].

According the prediction models reported by, Combe et al [2], for instance, variables reflecting disease activity also contribute to the prediction of joint damage. In this study, anti-CCP gave statistically significant differences between the positive and negative group with radiological damage. These data are comparable with the findings of other groups using a similar approach [15-17]. In our study serum anti-CCP added some prognostic information to ESR, CRP and RF, and it was the most sensitive predictor of joint damage, following ESR. These findings were in agreement with earlier reports [4], but other studies mentioned that anti-CCP was the only significant predictor, together with C reactive protein [18]. Vencovsky et al reported that the combination RF, CRP and anti-CCP2 antibodies revealed 10 times higher radiological damage [17] and early erosions [19]. However it was needed to be emphasized that the results based on dichotomization not useful in everyday clinical practice. So, we investigated whether appropriate cut off could be determined to identify those patients with the most aggressive radiological course.

Genetic studies have demonstrated that HLA alleles have been implicated in a number of chronic inflammatory diseases. RA has been associated with the SE of HLA-*DRB1*, which includes DRB1*04 and DRB1*01 alleles [19]. In the present study we found a discrepant relationship between RF and anti-CCP2 with the presence of the SE gene. Also our results do not support the opinion that there is a direct association between SE gene carriage and the occurrence of antibodies directed to CCP (or RFs) leading to the development of RA, but rather suggest that there is a synergistic interaction between these factors. In contrast Berglin et al reported that patients with early RA, had significant association between anti-CCP antibodies and expression of DRB1*0401/0101 [5]. The conversion of arginine to citrulline in HLA-DRB1*0401 transgenic mice has been demonstrated to significantly increase activation of CD4+ T cells [20], so that individuals carrying the SE genes may have more sustained T- and B-cell responses to citrullinated antigens than non carriers [13]. The presence of citrulline within the HLA binding peptide enhances the peptide-MHC affinity and leads to the activation of CD4+ T cells during presentation of citrullinated antigens [20]. This specific T-cell dependent immune response to citrullinated peptides may contribute to the occurrence of RA. A quite contradictory suggestion is that HLA antigens do not predispose to the autoimmune disease per se but rather fail to provide protection. Abnormal T-cell regulation associated with certain HLA haplotypes leads to the loss of self-tolerance followed by polyclonal activation of T and B cells and the subsequent production of auto antibodies [21].

In the present work there was a statistically significant difference observed between carriage of the DRB1*04 and DRB1*01 alleles with radiological damage and DRB1*04 with disease activity, these findings were in agreement with those previously reported [13,17]. Recent genome wide scanning studies using micro satellite loci have confirmed that there is strong linkage between this region with susceptibility and severity of RA [22,23]. Moreover Petersson et al [4] reported that DRB1*04 SE double gene dose is associated with disease severity in RA. In contrast Eberhardt et al [24] did not find the genotypes to be strongly associated with disease severity after two and five years.

The role of the SE in the evolution of articular destruction has yielded conflicting results; however, in most studies the presence of the SE is associated with increased joint destruction [14,22]. Others revealed an association between HLA-DRB1*04 and erosive disease [25], Goronzy et al [25], and Petersson et al [4] considered homozygosity for HLA-DRB1*04 as a major predictor of the development of erosions. In Caucasians of Northern European origin, the double dose of DRB1*04 SE alleles (0401/0401 genotype) was associated with radiographic signs of progressive joint damage [26]. A strong concordance was revealed between our study and that in East Asian populations, in which DRB1*0401 is rare and DRB1*0405 is the most frequent RA associated HLA-*DRB1 *genotype. The latter allele has also been reported to be associated with an increased risk for extra-articular manifestations of RA [27]. In contrast, others reported that the presence of shared epitope in single or double dose did not provide any predictive information on the development of joint damage and was not related to functional outcome [28]. In another study, by Mattey et al [16], reported that a predictive value of shared epitopes for radiological changes was restricted to RF negative patients. These discrepancies may reflect variability in the relative frequencies of HLA-DRB1*04 in different populations. Possibly, further studies on larger numbers of patients might solve this issue.

In the Present work the combination between anti-CCP, and the HLA-DR-B1*04 gave more identification of the grade of disease activity than a single test alone. The presence of RF, the SE, and anti-CCP2 antibodies yielded a 10 times higher average expectancy rate for a high radiological progression rate compared with the absence of the three parameters [15,16].

## Conclusion

Anti-CCP2 is superior to RF for the detection of RA and provided predictive information on joint destruction and disease activity. The presence of RA associated antibodies and/or the SE genes are indicative for a poorer radiological outcome and higher grade of activity. Thus, we shall need new strategies, both in research and in clinical practice, where we may now possess a new means for analyzing the risk of RA in individuals who will have different needs to acquire such information.

## Competing interests

The authors declare that they have no competing interests.

## Authors' contributions

NAF participated in study design, data acquisition and critical manuscript revision. AME-E participated in study design, performing laboratory investigations other than HLA, and critical manuscript revision. EM participated in study design, HLA analysis, analysis and interpretation of data, performed the statistical analysis, wrote the manuscript, and performing manuscript drafting and submission. RMB participated in study design, HLA analysis, analysis and interpretation of data, and critical manuscript revision. HBH participated in HLA analysis and manuscript revision. SA participated in study design, data acquisition, and critical manuscript revision. MM participated in study design and data acquisition. H-AGR participated in laboratory investigations other than HLA and manuscript revision. FA participated in study design, data acquisition, and critical manuscript revision. All authors read and approved the final manuscript.
